# Vector-Virus Mutualism Accelerates Population Increase of an Invasive Whitefly

**DOI:** 10.1371/journal.pone.0000182

**Published:** 2007-01-31

**Authors:** Min Jiu, Xue-Ping Zhou, Lin Tong, Jing Xu, Xiao Yang, Fang-Hao Wan, Shu-Sheng Liu

**Affiliations:** 1 Institute of Insect Sciences, Zhejiang University, Hangzhou, China; 2 Institute of Biotechnology, Zhejiang University, Hangzhou, China; 3 Institute of Plant Protection, Chinese Academy of Agricultural Sciences, Beijing, China; Massachusetts General Hospital and Harvard Medical School, United States of America

## Abstract

The relationships between plant viruses, their herbivore vectors and host plants can be beneficial, neutral, or antagonistic, depending on the species involved. This variation in relationships may affect the process of biological invasion and the displacement of indigenous species by invaders when the invasive and indigenous organisms occur with niche overlap but differ in the interactions. The notorious invasive B biotype of the whitefly complex *Bemisia tabaci* entered China in the late 1990s and is now the predominant or only biotype in many regions of the country. Tobacco curly shoot virus (TbCSV) and Tomato yellow leaf curl China virus (TYLCCNV) are two whitefly-transmitted begomoviruses that have become widespread recently in south China. We compared the performance of the invasive B and indigenous ZHJ1 whitefly biotypes on healthy, TbCSV-infected and TYLCCNV-infected tobacco plants. Compared to its performance on healthy plants, the invasive B biotype increased its fecundity and longevity by 12 and 6 fold when feeding on TbCSV-infected plants, and by 18 and 7 fold when feeding on TYLCCNV-infected plants. Population density of the B biotype on TbCSV- and TYLCCNV-infected plants reached 2 and 13 times that on healthy plants respectively in 56 days. In contrast, the indigenous ZHJ1 performed similarly on healthy and virus-infected plants. Virus-infection status of the whiteflies *per se* of both biotypes showed limited effects on performance of vectors on cotton, a nonhost plant of the viruses. The indirect mutualism between the B biotype whitefly and these viruses via their host plants, and the apparent lack of such mutualism for the indigenous whitefly, may contribute to the ability of the B whitefly biotype to invade, the displacement of indigenous whiteflies, and the disease pandemics of the viruses associated with this vector.

## Introduction

Plant-pathogen-vector systems are characterized by complex direct and indirect interactions [1], [2]. Direct interactions between the pathogen and the vector include transmission and dispersal of the pathogen by the vector, influence of the pathogen on the vector through its presence and replication within the latter, and sharing of the same host plant as food source between the pathogen and vector. Indirect interactions between the pathogen and the vector occur when one induces responses in the plant and alters the quality of the plant as a food source for the other. Both direct and indirect effects of the pathogen on the vector, or vice versa, can be beneficial or harmful, depending on the species [1]–[4]. For example, studies of the indirect effects of viruses on their vectors have revealed a variety of outcomes, ranging from positive [2], [5]–[10], neutral [11], [12], to negative [13], [14]. While the effects of these interactions on individual pathogens and herbivores are often quantified, the consequences of these interactions for the population dynamics of both types of organisms and the evolution of ecological communities have rarely been studied. For example, many invasive insects, such as some thrips, aphids, and mites, are effective virus vectors. Whether or not such vector-virus mutualisms have played a significant role in facilitating the success of invasion, and in some cases the displacement of indigenous species by the invaders is little understood.

The whitefly *Bemisia tabaci* (Gennadius) (Hemiptera: Aleyrodidae) is a genetically diverse group [15], [16]. More than 20 biotypes have been named from populations of this species complex [17], and more recently the populations have been grouped into five races plus an unresolved group [18]. The major feature of the global distribution of the numerous genetic types of *B. tabaci* is their strong geographic delineation [15], [16], [18], [19]. A clear exception is the B biotype [sometimes also referred to as *Bemisia argentifolii* (Bellows & Perring)] which in the past 20 years has spread rapidly around the world to become a major crop pest in tropical and subtropical regions [17], [20]–[23]. Damage by the pest occurs through phloem-feeding, excretion of honeydew, induction of phytotoxic disorders, and transmission of plant viruses [20], [24], [25]. The begomoviruses, the largest and most economically significant group of plant viruses [26], [27], are transmitted exclusively by *B. tabaci* in a persistent manner [28], [29].

While quantitative descriptions of the rapid and widespread invasion by the B biotype have rarely been reported, extensive circumstantial evidence from North, Central and South America as well as India has indicated co-occurrence of the invasion with two phenomena: disease pandemic caused by some begomoviruses and displacement of indigenous biotypes of the whitefly [10], [Bibr pone.0000182-Perring1] [Bibr pone.0000182-Brown1] [Bibr pone.0000182-Lima1] [Bibr pone.0000182-Seal1] [Bibr pone.0000182-Polston1] [Bibr pone.0000182-Ribeiro1] [32]. In China, local populations of *B. tabaci* have been recorded on various crops including tobacco since 1949 but the insect had never caused serious damage to any crops until the late 1990s when the B biotype invaded [33]–[35]. The B biotype is now the predominant or only biotype of *B. tabaci* in many regions in China [33]–[38]. Since the late 1990s, diseases caused by begomoviruses have also been increasing. In particular, two newly-characterized begomovirus species, Tobacco curly shoot virus (TbCSV) and Tomato yellow leaf curl China virus (TYLCCNV), have become widespread and caused extensive damage to tomato and tobacco crops in recent years in south China [39], [40]. Despite these extensive observations on the invasion of the B biotype whitefly in many regions of the world, the population expansion of the whitefly and the spread of begomovirus disease pandemic have largely been investigated separately. While increased populations of *B. tabaci* are often associated with the spread of plant-virus epidemics, the effects of vector-virus-plant interactions on the population expansion of the whitefly have been largely ignored [10].

In this study, we compared the performance of the invasive B biotype and an indigenous non-B (ZHJ1) biotype whitefly on healthy, TbCSV-infected and TYLCCNV-infected tobacco plants. We present evidence that the vector-virus-plant relationship differs between the notorious invasive B biotype and an indigenous biotype whitefly. While the invasive B biotype can achieve a much higher rate of reproduction on virus-infected plants than on healthy plants, the indigenous whitefly can not. We further show that the mutually beneficial relationships between the invasive whitefly and the plant viruses are indirect via the host plants. The whitefly achieved its improved performance through feeding on the virus-infected plants, but the presence of the viruses within the body of the whitefly has limited effect on the performance of the vector. This mutualism between the invasive B biotype whitefly and viruses, and the apparent lack of such mutualism for the indigenous whitefly, may have contributed to the invasion of B whitefly and the disease pandemics of the viruses associated with this vector in China and elsewhere.

## Results

### Fecundity and Longevity of Non-viruliferous Adult Whiteflies on Healthy or Virus-infected Tobacco

We first compared the fecundity and longevity of initially non-viruliferous whitefly adults, which had developed on cotton (a non-host plant of the two viruses) from egg to adulthood, on healthy and virus-infected plants. Adults were transferred onto the new plants upon emergence. We did the comparison for both the invasive B and the indigenous ZHJ1 whiteflies. All adults transferred onto either TbCSV-infected or TYLCCNV-infected plants became infected by the viruses after 48 h feeding [41].

Both whitefly biotype and plant health status had significant effects on fecundity and longevity, as did the biotype*plant interaction (Table 1). The B biotype had a higher fecundity, and lived longer, than the ZHJ1 biotype. More remarkably, in the B biotype, the mean number of eggs laid on TbCSV-infected or TYLCCNV-infected plants was twice as high as that on healthy plants, and the longevity was also 2–3 times longer. In contrast, adults of ZHJ1 had similar levels of fecundity and longevity on healthy and virus-infected plants (Table 1).

**Table 1:**
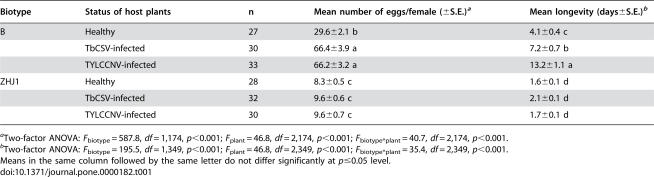
Fecundity and longevity of two biotypes of *Bemisia tabaci* females that developed on cotton from egg to adulthood and were transferred upon emergence onto healthy, TYLCCNV-infected or TbCSV-infected tobacco plants

Biotype	Status of host plants	n	Mean number of eggs/female (±S.E.)^a^	Mean longevity (days±S.E.)^b^
B	Healthy	27	29.6±2.1 b	4.1±0.4 c
	TbCSV-infected	30	66.4±3.9 a	7.2±0.7 b
	TYLCCNV-infected	33	66.2±3.2 a	13.2±1.1 a
ZHJ1	Healthy	28	8.3±0.5 c	1.6±0.1 d
	TbCSV-infected	32	9.6±0.6 c	2.1±0.1 d
	TYLCCNV-infected	30	9.6±0.7 c	1.7±0.1 d

a Two-factor ANOVA: *F*_biotype_ = 587.8, *df* = 1,174, *p*<0.001; *F*_plant_ = 46.8, *df* = 2,174, *p*<0.001; *F*_biotype*plant_ = 40.7, *df* = 2,174, *p*<0.001.

b Two-factor ANOVA: *F*_biotype_ = 195.5, *df* = 1,349, *p*<0.001; *F*_plant_ = 46.8, *df* = 2,349, *p*<0.001; *F*_biotype*plant_ = 35.4, *df* = 2,349, *p*<0.001.

Means in the same column followed by the same letter do not differ significantly at *p*≤0.05 level.

### Life-history Parameters of Whiteflies on Healthy and Virus-infected Tobacco

We next compared the performance of the two biotypes of whiteflies on healthy and virus-infected plants when they fed on the various plants from birth to death. The B biotype had a much higher level of survival from egg to adulthood than ZHJ1. Plant status did not affect survival of immature stages in either biotype (Table 2). Mean development time of the immature stages was not affected by whitefly biotype, but was significantly affected by plant status as well as by biotype*plant interaction (Table 2). Mean durations of development of the B biotype did not differ between the three types of plants, while development time of ZHJ1 was slightly reduced on TbCSV-infected plants and slightly increased on TYLCCNV-infected plants compared to that on healthy plants (Table 2).

**Table 2:**
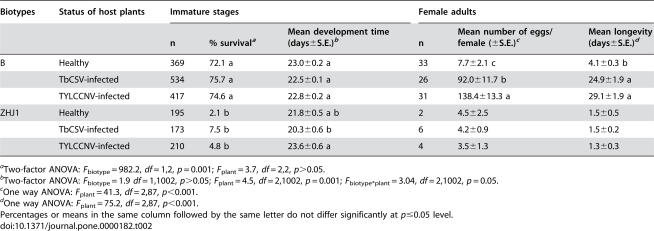
Performance of two biotypes of *Bemisia tabaci* on healthy, TYLCCNV-infected or TbCSV-infected tobacco plants

Biotypes	Status of host plants	Immature stages			Female adults		
		n	% survival^a^	Mean development time (days±S.E.)^b^	n	Mean number of eggs/female (±S.E.)^c^	Mean longevity (days±S.E.)^d^
B	Healthy	369	72.1 a	23.0±0.2 a	33	7.7±2.1 c	4.1±0.3 b
	TbCSV-infected	534	75.7 a	22.5±0.1 a	26	92.0±11.7 b	24.9±1.9 a
	TYLCCNV-infected	417	74.6 a	22.8±0.2 a	31	138.4±13.3 a	29.1±1.9 a
ZHJ1	Healthy	195	2.1 b	21.8±0.5 a b	2	4.5±2.5	1.5±0.5
	TbCSV-infected	173	7.5 b	20.3±0.6 b	6	4.2±0.9	1.5±0.2
	TYLCCNV-infected	210	4.8 b	23.6±0.6 a	4	3.5±1.3	1.3±0.3

a Two-factor ANOVA: *F*_biotype_ = 982.2, *df* = 1,2, *p* = 0.001; *F*_plant_ = 3.7, *df* = 2,2, *p*>0.05.

b Two-factor ANOVA: *F*_biotype_ = 1.9 *df* = 1,1002, *p*>0.05; *F*_plant_ = 4.5, *df* = 2,1002, *p* = 0.001; *F*_biotype*plant_ = 3.04, *df* = 2,1002, *p* = 0.05.

c One way ANOVA: *F*_plant_ = 41.3, *df* = 2,87, *p*<0.001.

d One way ANOVA: *F*_plant_ = 75.2, *df* = 2,87, *p*<0.001.

Percentages or means in the same column followed by the same letter do not differ significantly at *p*≤0.05 level.

Due to the low numbers of replicates with which we could assess fecundity and longevity in ZHJ1 on the three types of plants, these two parameters were not compared statistically between the two whitefly biotypes. The ZHJ1 generally had a lower fecundity and shorter longevity than B, while the plant status seemed to have little effect on both its fecundity and longevity (Table 2). In contrast, in the B biotype, the mean numbers of eggs laid on TbCSV-infected and TYLCCNV-infected plants were 12 and 18 times higher than that on healthy plants. Similarly, mean longevity of the B biotype on TbCSV-infected and TYLCCNV-infected plants was 6 and 7 times longer than that on healthy plants (Table 2).

### Population Increase of B biotype Whitefly on Healthy and Virus-infected Tobacco

We then compared the population increase of the B biotype whitefly on healthy, TbCSV-infected and TYLCCNV-infected plants. Plants in the three treatments were grown to bear 9–10 true leaves by the 28^th^ day and to bear 12–13 true leaves by the 56^th^ day after *B. tabaci* adults were released onto them. All plants grew well although TbCSV-infected and TYLCCNV-infected plants showed the characteristic disease symptoms.

**Figure 1:**
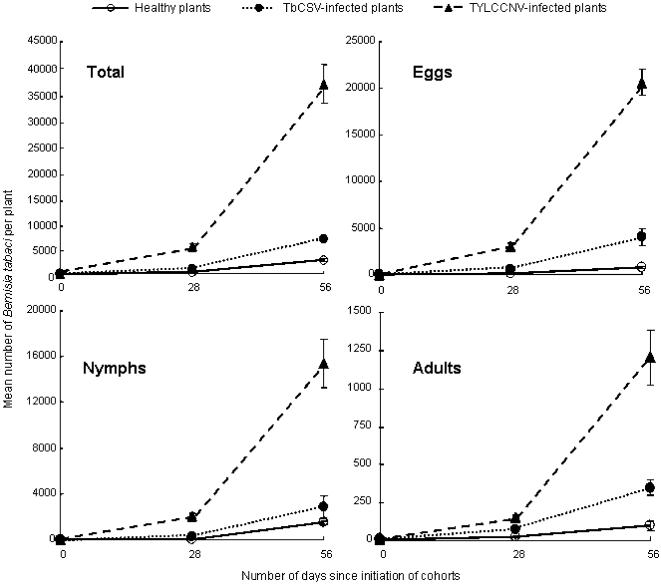
Mean numbers (±standard error) of eggs, nymphs, adults or all individuals per plant at two sampling dates of three cohorts of the B biotype *Bemisia tabaci* that were initiated on healthy, TbCSV-infected, or TYLCCNV-infected tobacco plants. Each plant was inoculated with 5 female and 5 male adult whiteflies.

By the 28^th^ day, the mean number of whiteflies per plant on healthy plants reached 401. The mean number on TbCSV-infected and TYLCCNV-infected plants was 1.9 and 10.8 times higher than that on healthy plants, and such a difference was evident for all life stages (Table 3, Figure 1). By the 56^th^ day, the mean number of whiteflies per plant on healthy plants reached 2340. The mean number on TbCSV-infected and TYLCCNV-infected plants was 2.1 and 12.8 times higher than that on healthy plants, and such a difference was also evident for all life stages (Table 3, Figure 1).

**Table 3:**
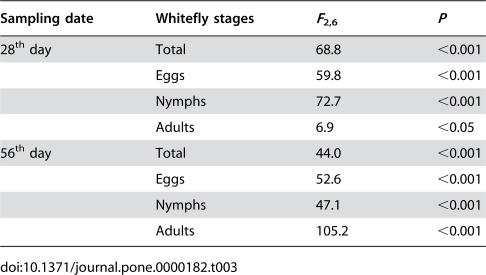
One-way ANOVA statistics for analyzing Ln(number+1) of whiteflies on healthy, TbCSV-infected and TYLCCNV-infected tobacco plants

Sampling date	Whitefly stages	*F*_2,6_	*P*
28^th^ day	Total	68.8	<0.001
	Eggs	59.8	<0.001
	Nymphs	72.7	<0.001
	Adults	6.9	<0.05
56^th^ day	Total	44.0	<0.001
	Eggs	52.6	<0.001
	Nymphs	47.1	<0.001
	Adults	105.2	<0.001

### Life-history Parameters of Non-viruliferous and Viruliferous Whiteflies on Cotton

Finally, we compared the performance of non-viruliferous, TbCSV-infected and TYYLCCNV-infected whiteflies on cotton, a nonhost plant of the two viruses, to examine the effects of the direct association between the vectors and viruses. We did the comparison for both biotypes of whiteflies. Both whitefly biotype and whitefly virus-infection status had significant effects on fecundity and longevity; the biotype*status interaction had a significant effect on fecundity but not on longevity (Table 4). In the B biotype, fecundity of TbCSV-infected adults was significantly increased while both fecundity and longevity of TYLCCNV-infected adults were reduced, compared to non-viruliferous adults (Table 4). In ZHJ1, fecundity and longevity of TbCSV-infected adults remained unchanged, while those of TYLCCNV-infected adults were significantly reduced, compared to non-viruliferous adults (Table 4).

**Table 4:**
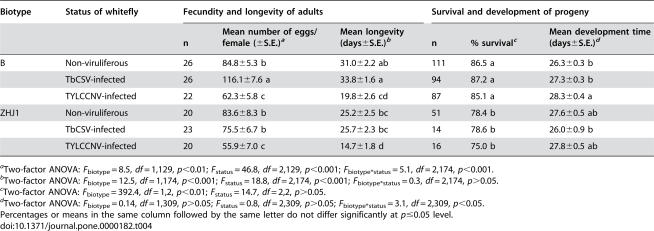
Fecundity and longevity of non-viruliferous and viruliferous females of two biotypes of *Bemisia tabaci*, as well as survival and development time of the progeny produced by these females, on healthy cotton

Biotype	Status of whitefly	Fecundity and longevity of adults			Survival and development of progeny		
		n	Mean number of eggs/female (±S.E.)^a^	Mean longevity (days±S.E.)^b^	n	% survival^c^	Mean development time (days±S.E.)^d^
B	Non-viruliferous	26	84.8±5.3 b	31.0±2.2 ab	111	86.5 a	26.3±0.3 b
	TbCSV-infected	26	116.1±7.6 a	33.8±1.6 a	94	87.2 a	27.3±0.3 b
	TYLCCNV-infected	22	62.3±5.8 c	19.8±2.6 cd	87	85.1 a	28.3±0.4 a
ZHJ1	Non-viruliferous	20	83.6±8.3 b	25.2±2.5 bc	51	78.4 b	27.6±0.5 ab
	TbCSV-infected	23	75.5±6.7 b	25.7±2.3 bc	14	78.6 b	26.0±0.9 b
	TYLCCNV-infected	20	55.9±7.0 c	14.7±1.8 d	16	75.0 b	27.8±0.5 ab

a Two-factor ANOVA: *F*_biotype_ = 8.5, *df* = 1,129, *p*<0.01; *F*_status_ = 46.8, *df* = 2,129, *p*<0.001; *F*_biotype*status_ = 5.1, *df* = 2,174, *p*<0.001.

b Two-factor ANOVA: *F*_biotype_ = 12.5, *df* = 1,174, *p*<0.001; *F*_status_ = 18.8, *df* = 2,174, *p*<0.001; *F*_biotype*status_ = 0.3, *df* = 2,174, *p*>0.05.

c Two-factor ANOVA: *F*_biotype_ = 392.4, *df* = 1,2, *p*<0.01; *F*_status_ = 14.7, *df* = 2,2, *p*>0.05.

d Two-factor ANOVA: *F*_biotype_ = 0.14, *df* = 1,309, *p*>0.05; *F*_status_ = 0.8, *df* = 2,309, *p*>0.05; *F*_biotype*status_ = 3.1, *df* = 2,309, *p*<0.05.

Percentages or means in the same column followed by the same letter do not differ significantly at *p*≤0.05 level.

The percentage survival from egg to adulthood of the B biotype was significantly higher than that of ZHJ1, but the survival was not affected by infection status of whitefly in either biotype (Table 4). Development time was not affected by either whitefly biotype or whitefly infection status, but was significantly affected by biotype*status interaction. The only noticeable effect was that the development time of progeny produced by TYLCCNV-infected adults was significantly longer than that of progeny produced by non-viruliferous adults (Table 4).

## Discussion

In comparison with its performance on healthy tobacco, the B biotype experienced a significant increase in its fecundity and longevity through feeding on plants infected with the two begomoviruses. The benefits were quickly achieved by non-viruliferous whitefly adults when they were transferred from a nonhost plant of the viruses to virus-infected plants (Table 1). The benefits were more substantial when the whitefly completed its life cycle on the virus-infected plants (Table 2). These benefits were shown to speed up population increase by 2 or 12 fold when the host plants were infected with TbCSV or TYLCCNV, respectively (Figure 1). However, the presence of TbCSV within the B whitefly increased fecundity of the vector by only 36% and had no effects on longevity of adults and survival and development time of the progeny, while the presence of TYLCCNV within the B whitefly actually reduced fecundity and longevity of the vector by 27% and 36%. Therefore, the B biotype acquired the benefits mainly through indirect mutualism with the viruses, particularly TYLCCNV, via their shared host plants.

In contrast, the performance of the indigenous ZHJ1 biotype whitefly was similar on healthy and virus-infected plants, whether non-viruliferous adults were transferred onto virus-infected plants (Table 1) or the whitefly completed its life cycle on the virus-infected plants (Table 2). The population increases of ZHJ1 on healthy and virus-infected plants were not compared in the current study due to lack of resources at the time when the experiments were performed. Population increase of ZHJ1 under caged conditions has been investigated on host plants of different levels of suitability [37], [32], [42]. The rate of population increase of this biotype is always positively correlated with its performance on undetached leaves in clip-cages (37, unpublished data). It is likely that population increases on healthy and virus-infected tobacco plants would be similar as indicated by its similar performance on undetached leaves of virus-infected and healthy tobacco plants (Tables 1 and 2).

Both B biotype and ZHJ1 whiteflies are effective vectors of TbCSV and TYLCCNV [41], and both of them have been recorded from a number of host plants such as tobacco, tomato, cotton, sweet potato, squash, egg plant and soybean in the field (Xu J and Liu SS, unpublished data). However, the two biotypes are reproductively isolated [43], and the B biotype has a wider host range than ZHJ1 and generally performs better on their shared host plants [42]. In the laboratory, Zang et al. [37] demonstrated that the B biotype had the capacity to displace ZHJ1 on plants that were equally suitable to both of them, and the speed of displacement progressed faster as the relative suitability of the host plant for ZHJ1 decreased. Apparently, the wider host range of the B biotype over that of ZHJ1 gives it a competitive advantage, although the mechanisms for the competitive advantage of the B biotype over the indigenous biotype, in the absence of plant viruses, are still under investigation. The differential vector-virus relationships between the two biotypes observed in this study indicates that the B biotype has a significant advantage via its mutualism with the viruses to increase the suitability of host plants for its population increase, while the indigenous biotype is unable to do so. Tobacco is a relatively poor host plant for both whitefly biotypes used in this study, especially for ZHJ1, although the suitability of this plant species differs in degree among cultivars [42], [44] (Tables 1 and 2). However, the B biotype is able to transmit the two begomoviruses into this host plant and then increase its reproduction substantially on the virus-infected plants as compared to that on plants uninfected by the viruses (Tables 1 and 2, Figure 1). In contrast, the indigenous ZHJ1 whitefly is unable to increase its reproduction on virus-infected plants, although it can transmit the two viruses as effectively as the B whitefly [41].

To our knowledge, this is the first study to demonstrate that two biotypes of an insect species complex, one invasive and the other indigenous, differ markedly in their relationships with the viruses they transmit. The invasive B biotype of the whitefly, in contrast to the indigenous ZHJ1 biotype, benefits from infection of host plants by the viruses. The tobacco cultivar used in this study, NC89, has been widely cultivated in regions of south China, where pandemics of TbCSV and TYLCCNV have been occurring on tobacco crops since the invasion of the B biotype in the late 1990s [39], [40], and local biotypes of *B. tabaci* are no longer found in recent collections in the past two years (Xu J and Liu SS, unpublished data). The circumstantial evidence suggests that the comparative advantage of the B biotypes over local biotypes acquired through its mutualism with the viruses may have assisted its invasion and displacement of the local biotypes in many regions of south China. Other circumstantial evidence indicates that this situation may not be unique. In the USA, McKenzie et al. [8] and McKenzie [45] demonstrated that the invasive B biotype whitefly had higher fecundity and increased faster on tomato plants infected with tomato mottle virus than on healthy plants, while Costa et al. [46] showed that the indigenous A biotype (not detailed in the original article, but identity of the biotype was confirmed subsequently, J. K. Brown, personal communication) exhibited generally similar or lower levels of survival and fecundity on six species of host plants infected with various viruses compared to its performance on healthy plants. It is possible that the B biotype also has an advantage in its mutualism with begomoviruses over the A biotype in the USA, and this advantage has assisted in its apparently rapid invasion and displacement of the indigenous A biotype in that country [13], [32], [47]. In south India, the B biotype whitefly was first reported in 1999 and has since been spreading; the B biotype has become more common than the indigenous biotypes in places where it has been present for more than 2 years, and the invasion of the B biotype has been associated with the appearance of new epidemics of various viruses vectored by *B. tabaci* [10], [23]. It is also likely that other invasive insects, such as the western flower thrips, *Frankliniella occidentalis*, may have taken the advantage of mutualism with the viruses it transmits in its widespread invasion [1], [9], [48].

In plant-pathogen-vector systems such as the one examined in this study, the pathogen depends on the arthropod herbivore vector for transmission and dispersal. Thus higher rates of vector population increase through mutualism with the viruses will in turn facilitate the spread of virus disease pandemic. Laboratory and field evidence indicates that this kind of vector-virus mutualism has played a significant role in driving the spread of a cassava mosaic disease pandemic in Uganda [10], [49]. Circumstantial evidence for this kind of vector-virus mutualism in assisting in the spread of virus disease pandemic has been seen in other plant-vector-systems, such as some aphid-transmitted viruses in wheat, barley and oats [6], [7], [50].

Since the effect of plant virus on the vector can vary from positive to negative, effort has been made to reveal patterns of the effects according to the type of plant-virus-vector relationships. Studies on virus-thrips interactions suggest that an indirect positive effect of virus infection of host plant on the vector is more likely to occur on plants of poor quality [9]. A study on performance of the aphid *Myzus persicae* on potato plants infected with different viruses indicates that improved fitness of the vector is found only where the relationship between the vector and the virus is close and the virus is transmitted in a persistent manner [51]. While indications from both these studies [9], [51] seem consistent with the results obtained in our study, since tobacco is a relatively poor host and both TbCSV and TYLCCNV are transmitted by the whitefly in a persistent (circulative, non-propagative) manner [39], [40], they offer no explanation for the differential interactions in the B biotype and ZHJ1 whiteflies.

Improved performance of sucking insects on virus-infected plants is often correlated with increases in free amino acids in the plants [2]. This correlation has been shown for the African cassava *B. tabaci* on cassava plants infected with the Cassava mosaic viruses [10]. Whether the increased fitness of the B biotype whitefly on TbCSV- or TYLCCNV-infected tobacco involves increase of free amino acids in the plants remain to be investigated. Another possible mechanism for a positive effect of viruses on the vector via host plants is a virus-induced suppression of plant defence against the herbivore [1].

As viruses and their vectors compete for the same host plants as a shared food source, the vector-virus beneficial mutualism may present an evolutionary dead end for the plant-virus-vector systems. Plants should have evolved a balanced system of defence against multiple biotic threats [2]. However, many crop cultivars have been genetically altered in comparison to their ancestral stocks and may have defence systems that differ from those in naturally co-evolved plants. Crops such as tomato and tobacco are planted in enormous acreages every year, and there is always an abundance of food source for both partners. The challenge, as always, remains for us to gain a deeper insight into the plant-pathogen-vector relationships, for a better understanding of the ecological or evolutionary processes underlying the invasion of vector herbivores and pandemic of virus diseases they transmit, and so improve their management.

## Materials and methods

### Whiteflies

Two biotypes of the whitefly species complex *Bemisia tabaci* were used. The B biotype population was first collected from cabbage, *Brassica oleracea* var. *capitata* L. (Cruciferae), and the non-B (ZHJ1) population from cotton, *Gossypium hirsutum*, in 2003 in Hangzhou, Zhejiang, China (32.2°N, 120.1°E, with an elevation of 6 m a.s.l.). The B biotype (GenBank Acc. No. AJ867555) was shown to be exotic to China, and ZHJ1 (GenBank Acc. No. AJ867556) is an indigenous non-B population [43], [52]. ZHJ1 belongs to the unresolved Asia group of *B. tabaci* as described by De Barro et al. [18]. In Zhejiang, ZHJ1 has been found to occur on cotton, tobacco, tomato, sweet potato, squash, eggplant, and soybean [52] (Xu J and Liu SS, unpublished data; GenBank Acc. No. DQ309074). A search of the data in GenBank indicated that whitefly populations that share over 99% similarity of COI sequences with ZHJ1 also occur in Jiangsu and Guangzhou, China (GenBank Acc. No. AY686088 and AY686083, respectively). Stock cultures of the two biotypes were maintained on cotton *G. hirsutum* L. cv. Chuanmian 109 in separate climate chambers at 28±1°C, 14 h light∶10 h darkness and 70±10% r.h. The purity of the cultures was monitored every 3–5 generations using the random amplified polymorphic DNA-polymerase chain reaction (RAPD-PCR) technique [43], and measures were taken to use only pure sub-cultures of the respective biotypes for experiments.

### Viruses

Infectious clones of TbCSV and TYLCCNV and their satellite DNA molecules (named DNAβ) constructed previously [39], [40] were used as inocula, and the viruses were maintained on plants of tobacco *Nicotiana tabacum* L. cv. NC89.

### Plants

Tobacco *Nicotiana tabacum* L. cv. NC89, a host plant of TbCSV and TYLCCNV, and cotton (cv. Chuanmian 109), a non-host plant of TbCSV and TYLCCNV, were used. Uninfected tobacco and cotton plants were grown in a potting mix in 11 cm plastic pots in insect-proof cages under natural lighting and ambient temperature in screen houses. To obtain virus-infected tobacco, the plants at the 4–5 true-leaf stage were inoculated with TbCSV and its DNAβ or TYLCCNV and its DNAβ by agroinoculation as previously described [39], [40], [53]. Healthy, TbCSV-infected or TYLCCNV-infected tobacco plants were grown to the 6–7 true-leaf stage for experiments. Virus infection of test plants was judged by the appearance of characteristic symptoms caused by each of the two viruses [39], [40] and further confirmed by molecular techniques as described below. Cotton plants were also grown to the 6–7 true-leaf stage for experiments. All plants were watered every 3–4 days as necessary and fertilized once a week.

All experiments were conducted at 26±1°C, 40–60% relative humidity, and a photoperiod of 14 h light∶10 h darkness.

### Detection of TbCSV and TYLCCNV DNA

Nucleic acids from individual whiteflies and plants were extracted using the methods of Luo et al. [33] and Xie et al. [54], respectively. TbCSV and its associated DNAβ or TYLCCNV and its associated DNAβ in individual whiteflies and infected plants were detected using the primers and PCR procedures as described by Cui et al. [39] and Li et al. [40].

### Fecundity and longevity of non-viruliferous adult whiteflies on healthy or virus-infected tobacco

These experiments were conducted to examine the fecundity and longevity of adult whiteflies that were reared on cotton from egg to adulthood and then transferred upon emergence onto healthy or virus-infected tobacco plants. The experiments for the B and ZHJ1 biotypes were carried out concurrently using the following procedure. For each biotype, approximately 300 newly emerged (0–24 h) adult whiteflies were collected from the culture on cotton and divided randomly into three groups of about 100 each, to be used for inoculation onto the three types of tobacco plants: healthy, TbCSV-infected or TYLCCNV-infected. On each type of plants, approximately 30 replicates were conducted. In each replicate on a given type of plants, one female and one male adults were placed on the lower surface of a plant leaf (third to fifth leaf from the top) enclosed in a clip-cage. This ventilated clip-cage was made from a clear plastic cup, a metal clip, and white plastic mesh, and measures 30 mm in diameter, 30 mm in height, and 5 g in weight [55]. Every 3 days, the leaves bearing the adults were examined using a dissecting microscope to count the number of eggs laid, and the adults were transferred to new leaves, until death of the females.

The experiments include two whitefly biotypes and three types of plants, making up six treatments (Table 1). For analysis of the performance of the two biotypes on the three types of plants, a two-factor analysis of variance (ANOVA) was performed. The two factors were biotype (two levels) and plant status (three levels). The two response variables of fecundity and longevity were analyzed individually (Table 1). When an overall ANOVA indicated significant effects of the factors or their interactions at *P*<0.05, the means were compared using a Tukey test. All statistical analyses were done using the statistical software, BIOMstat (version 3.30q) [56].

### Life-history parameters of whiteflies on healthy and virus-infected tobacco

Six treatments, composed of two whitefly biotypes and three types of plants, were conducted (Table 2). For each of the six treatments, approximately 300 adults, 4–6 days post emergence, were collected from the culture on cotton and released onto a tobacco plant. The adults were left on the plant for 3 h to oviposit and then removed. The eggs laid for each treatment were counted using a microscope. From the 15^th^ day onwards, daily observations were made to collect and record newly emerged adults until all pupae had emerged or died. From these data we calculated the percentage survival and development time from egg to adulthood.

During adult emergence in each treatment, pairs of newly-eclosed adults, one male and one female in each pair, were collected and placed onto leaves (second to fourth leaves from the top) of the same type of plant using clip cages, to observe their fecundity and longevity. Every 3 days, the leaves bearing the adults were examined using a dissecting microscope to count the number of eggs laid, and the adults were transferred to new leaves until deaths of the females. For the B biotype, approximately 30 replicates were conducted for each of the three treatments, while for the ZHJ1 biotype a lower number of replicates were conducted due to the low numbers of females that were available (Table 2).

The data of percentage survival were transformed by arcsine square root before ANOVA. For analysis of the percentage survival or mean development time for the two whitefly biotypes and three types of plants, a two-factor ANOVA was conducted as described above. For the analysis of fecundity and longevity of the B biotype on the three types of plants, a one-way ANOVA was performed (Table 2).

### Population increase of B biotype whitefly on healthy and virus-infected tobacco

Three treatments of population development of the B biotype whitefly on healthy, TbCSV-infected and TYLCCNV-infected tobacco plants were conducted. Ninety newly emerged (0–24 h) female and 90 newly emerged male adult whiteflies were collected from the culture on cotton and divided randomly into 18 groups, each consisting of 5 females and 5 males. The 18 groups of adults were inoculated onto 18 tobacco plants: 6 healthy, 6 TbCSV-infected and 6 TYLCCNV-infected, making up 6 replicates for each of the three treatments. The 18 plants were all at the 6–7 true-leaf stage and placed individually in whitefly-proof, ventilated cages (55 cm×55 cm×55 cm), in one room at 26±1°C, 40–60% relative humidity, and a photoperiod of 14 h light : 10 h darkness. On the 28^th^ day after inoculation, three plants from each of the three treatments were sampled to count all eggs, nymphs and adults in each of the replicates. The remaining three plants in each of the three treatments were likewise sampled on the 56^th^ day.

Since the mean number per plant in each of the three treatments on the 56^th^ day was obviously much higher than that on the 28^th^ day (Figure 1), no comparison was considered between the two sampling dates. Mean numbers of whiteflies on the three types of plants were analyzed using one-way ANOVA for each of two sampling dates. Ln(number+1) was performed to transform the numbers of eggs, nymphs, adults and all individuals on each plant to be used for ANOVA (Table 3).

### Life-history parameters of non-viruliferous and viruliferous whiteflies on cotton

Six treatments, composed of two biotypes and three categories of status of whiteflies, were conducted (Table 4). For the observations on fecundity and longevity of non-viruliferous and viruliferous adults in each of the two biotypes, approximately 200 newly emerged adult whiteflies were collected from the culture on cotton and divided randomly into three groups of 60–70 each, to be used for inoculation onto the three types of tobacco plants: healthy, TbCSV-infected or TYLCCNV-infected. The three groups of adults were left to feed on the three types of plants for 48 h, and then collected to initiate three treatments on cotton (Table 4). By the end of the 48 h, the two groups of adults feeding on TbCSV-infected or TYLCCNV-infected plants would all have become viruliferous [41]. In each of the three treatments, 25–30 replicates were conducted. In each replicate, one female and one male were placed on the lower surface of a plant leaf (third to fifth leaf from the top) enclosed in a clip-cage. Every 3 days, the leaves bearing the adults were examined using a dissecting microscope to count the number of eggs laid, and the adults were transferred to new leaves, until death of the females.

For observations on the survival and development time of progeny produced by non-viruliferous and viruliferous adults in each of the two biotypes, three groups of non-viruliferous, TbCSV-infected and TYLCCNV-infected adults were again obtained using the procedure described above. The three groups of adults were each released onto a cotton plant in a whitefly-proof cage, left there to oviposit for 24 h and then removed. The eggs laid on each plant, i.e., in each treatment, were counted using a microscope. From the 15^th^ day onwards, daily observations were made to collect and record newly emerged adults, until all pupae had emerged or died. From these data we calculated the percentage survival and development time from egg to adulthood.

The data of percentage survival were transformed by arcsine square root before ANOVA. For analysis of each of the four response variables: fecundity, adult longevity, survival of progeny, and development time of progeny, a two-factor ANOVA was conducted, and the two factors were whitefly biotype (two levels) and whitefly status (three levels) (Table 4). When an overall ANOVA indicated significant effects of the factors or their interactions at *P*<0.05, the means were compared using a Tukey test.
